# Autosomal genetic control of human gene expression does not differ across the sexes

**DOI:** 10.1186/s13059-016-1111-0

**Published:** 2016-12-01

**Authors:** Irfahan Kassam, Luke Lloyd-Jones, Alexander Holloway, Kerrin S. Small, Biao Zeng, Andrew Bakshi, Andres Metspalu, Greg Gibson, Tim D. Spector, Tonu Esko, Grant W. Montgomery, Joseph E. Powell, Jian Yang, Peter M. Visscher, Allan F. McRae

**Affiliations:** 1 Queensland Brain Institute, The University of Queensland, Brisbane, Australia; 2 Department of Twin Research and Genetic Epidemiology, King’s College London, London, UK; 3 School of Biology and Centre for Integrative Genomics, Georgia Institute of Technology, Atlanta, USA; 4 Estonian Genome Centre, University of Tartu, Tartu, Estonia; 5 Institute for Molecular Bioscience, University of Queensland, Brisbane, Australia; 6 QIMR Berghofer Medical Research Institute, Brisbane, Australia; 7 University of Queensland Diamantina Institute, Translational Research Institute, The University of Queensland, Brisbane, Australia

**Keywords:** Gene expression, Genetic correlation, Sex-specific genetic architecture

## Abstract

**Background:**

Despite their nearly identical genomes, males and females differ in risk, incidence, prevalence, severity and age-at-onset of many diseases. Sexual dimorphism is also seen in human autosomal gene expression, and has largely been explored by examining the contribution of genotype-by-sex interactions to variation in gene expression.

**Results:**

In this study, we use data from a mixture of pedigree and unrelated individuals with verified European ancestry to investigate the sex-specific genetic architecture of gene expression measured in whole blood across *n*=1048 males and *n*=1005 females by treating gene expression intensities in the sexes as two distinct traits and estimating the genetic correlation (*r*_G_) between them. These correlations measure the similarity of the combined additive genetic effects of all single-nucleotide polymorphisms across the autosomal chromosomes, and thus the level of common genetic control of gene expression across the sexes. Genetic correlations are estimated across the sexes for the expression levels of 12,528 autosomal gene expression probes using bivariate GREML, and tested for differences in autosomal genetic control of gene expression across the sexes. Overall, no deviation of the distribution of test statistics is observed from that expected under the null hypothesis of a common autosomal genetic architecture for gene expression across the sexes.

**Conclusions:**

These results suggest that males and females share the same common genetic control of gene expression.

**Electronic supplementary material:**

The online version of this article (doi:10.1186/s13059-016-1111-0) contains supplementary material, which is available to authorized users.

## Background

Despite their nearly identical genomes [1], males and females differ in risk, incidence, prevalence, severity and age-at-onset of many diseases including autoimmune diseases [2], cancers [3, 4], cardiovascular diseases [5], and neurological and psychiatric disorders [6–9]. It has been postulated that humans have a sex-specific genetic architecture, where for example, dosage differences in X-linked genes are thought to account for some of the sex-specific genetic architecture of phenotypes, and that the autosomal contribution of phenotypic differences across the sexes is due to differences in the regulation of genes rather than the gene content [10]. Motivated by observing large mean differences in phenotypes across the sexes, studies investigating the sex-specific genetic architecture of phenotypes typically have used a sex-stratified genome-wide association study (GWAS) or genotype-by-sex interaction approach [11, 12].

Recent studies have examined the sex-specific autosomal genetic architecture of high-level human complex traits by treating them as two distinct traits for males and females, and estimating the autosomal genetic correlation across the sexes [13, 14]. The genetic correlation between two traits is a measure of the common segregating genetic variants causing simultaneous variation in both traits, and by definition ranges from −1 to 1 [15]. The degree of genetic correlation expresses the extent to which these two traits are influenced by the same genetic variants and, in theory, represents the combined additive genetic effects of all causal loci across the genome, and thus the level of common genetic control; in practice, however, where DNA microarray data are used to tag genetic variants assumed to be in linkage disequilibrium with unknown causal variants, the genetic correlation represents the aggregate genetic effect of all tagged genetic variants across the genome. The genetic correlations of these high-level human complex traits were found to be large and positive, indicating that the additive genetic effects of all genetic variants across the autosomes have the same effect on these phenotypes in both sexes. Similar results have been observed in other species, where the majority of genetic correlation estimates were found to be large and positive, and rare cases where estimates are negative were related to fitness [16].

Gene expression can be thought of as a low level or intermediate trait and can be used to help understand the genetic and molecular basis for phenotypic differences across sexes. Like the study of high-level human complex traits, the sex-specific genetic architecture of gene expression in humans has largely been explored by examining the contribution of genotype-by-sex interactions to variation in gene expression (i.e. sex-specific eQTLs) [17–19]. Overall, there has been weak evidence for sex-specific eQTLs, which range from claims that 15% of detected *cis*-eQTLs show sex-specific effects in lymphoblastoid cell lines [17], to six autosomal and X chromosome genes showing sex-specific eQTLs in whole blood [19], to zero detected sex-specific eQTLs in cerebellar and frontal cortex brain tissue [18]. Potential reasons for these observations are: (1) the contribution of genotype-by-sex interactions to variation in gene expression may be tissue-specific, since sexually dimorphic genes have shown tissue-specific patterns [20–22]; (2) without sufficiently large sample sizes, the power to detect sex-specific eQTLs can be low, since there would need to be a correction to the significance threshold to account for both the number of genetic variants and the number of genes tested; or (3) the contribution of genotype-by-sex interactions to variation in gene expression occurs in a small number of genes, and, on average, males and females share the same common genetic control of gene expression.

In this study, we examine the sex-specific genetic architecture of gene expression measured in whole blood by estimating the genetic correlation (*r*_G_) of 12,528 autosomal gene expression probes across *n*=1048 males and *n*=1005 females. By treating gene expression intensities in the sexes as two distinct traits, we estimate the combined additive genetic effects of all single-nucleotide polymorphisms (SNPs) across the autosomal chromosomes, and thus the level of common genetic control of gene expression across the sexes. In this context, *r*_G_=1 means that males and females share the same common genetic control of gene expression, while any *r*_G_<1 indicates that the genetic control of gene expression differs across the sexes.

## Results

Gene expression and genotype data were available from a concatenated dataset of *n*=2053 pedigree and unrelated individuals from three distinct cohorts with verified European ancestry (see ‘Methods’). Gene expression probes were restricted to those with estimated heritability greater than 10%, since estimating the genetic correlation of gene expression across the sexes requires a heritable component in both sexes (see ‘Methods’). We first confirmed extensive sexually dimorphic gene expression in 12,528 autosomal gene expression probes across *n*=1048 males and *n*=1005 females. A total of 1413 autosomal probes corresponding to 1266 unique genes showed significant mean differences in expression intensities across the sexes at a Bonferroni corrected threshold of *P*=3.99×10^−6^ (Additional file 1: Figure S1). The proportion of these probes showing higher expression in one sex over the other was approximately even, with 50.5% of these probes (713 of the 1413 probes) showing higher expression in females compared to males.

**Fig. 1:**
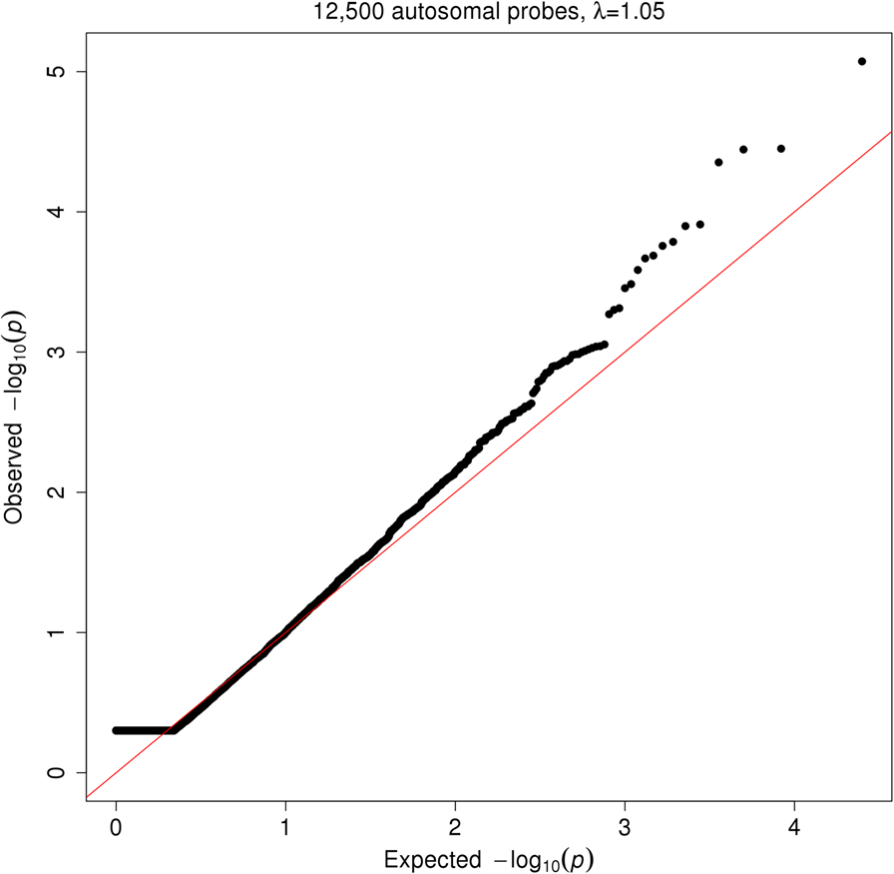
Quantile–quantile plot for expected versus observed *P* values from a test of genetic correlation of 12,500 genome-wide gene expression probes across males and females. The distribution of *P* values is initially flat for values of 0.5, which is attributed to the likelihood ratio test being on the edge of the parameter space. Subsequently, the distribution closely follows the null distribution with little deviation of the test statistics from the expected

**Fig. 2:**
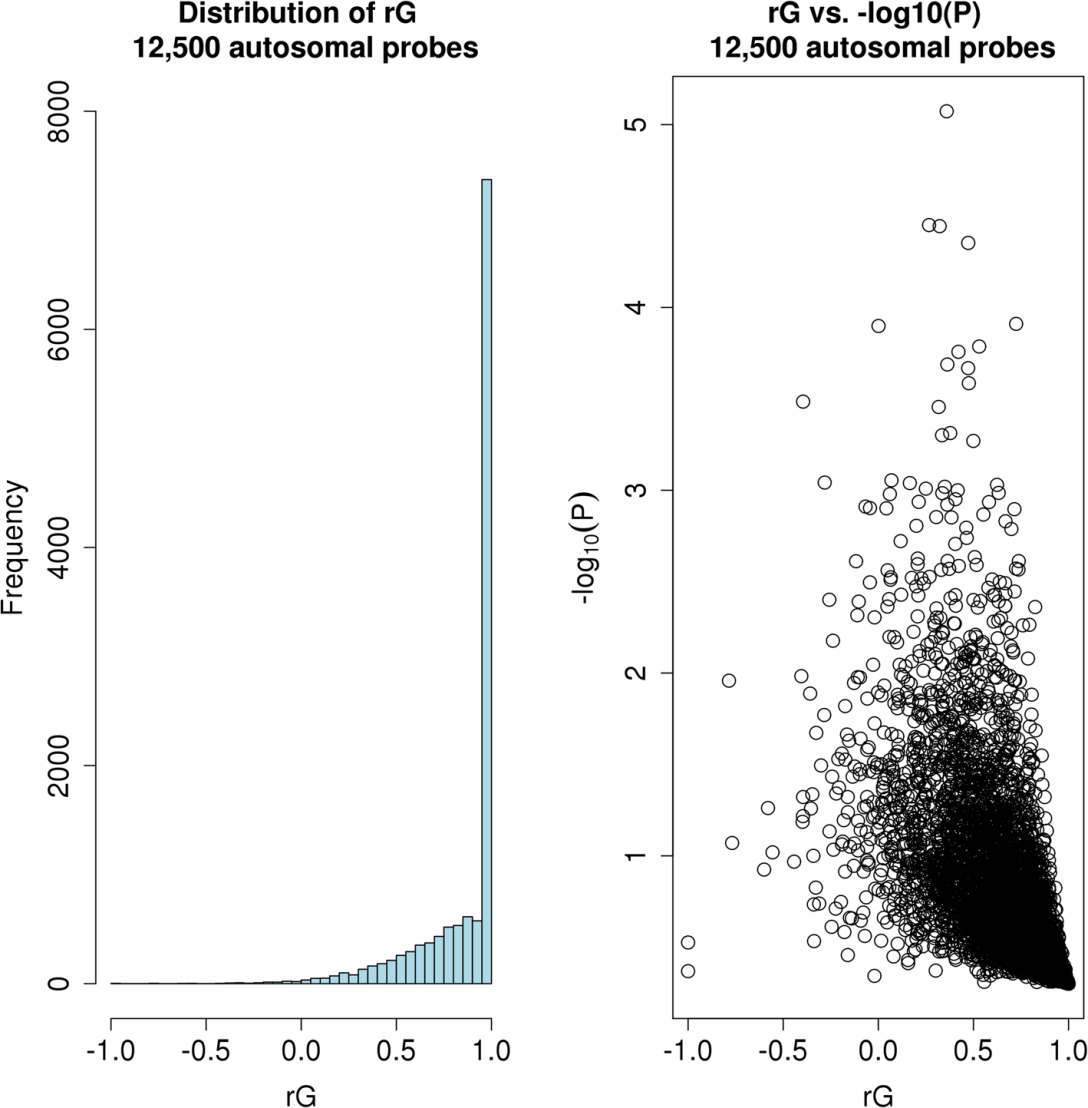
Distribution of 12,500 estimated genetic correlations of gene expression across males and females from a bivariate GREML analysis. *Left*: The distribution of the estimated *r*_G_ is skewed towards *r*_G_=1.00, with a large peak at values close to *r*_G_=1.00. The median estimate across all tested probes is *r*_G_=1.00 indicating that, on average, males and females share the same common genetic control of gene expression. *Right*: The estimated *r*_G_ compared to their corresponding *P* value. No probes satisfied the Bonferroni corrected significance threshold of *P*=3.99×10^−6^

**Fig. 3:**
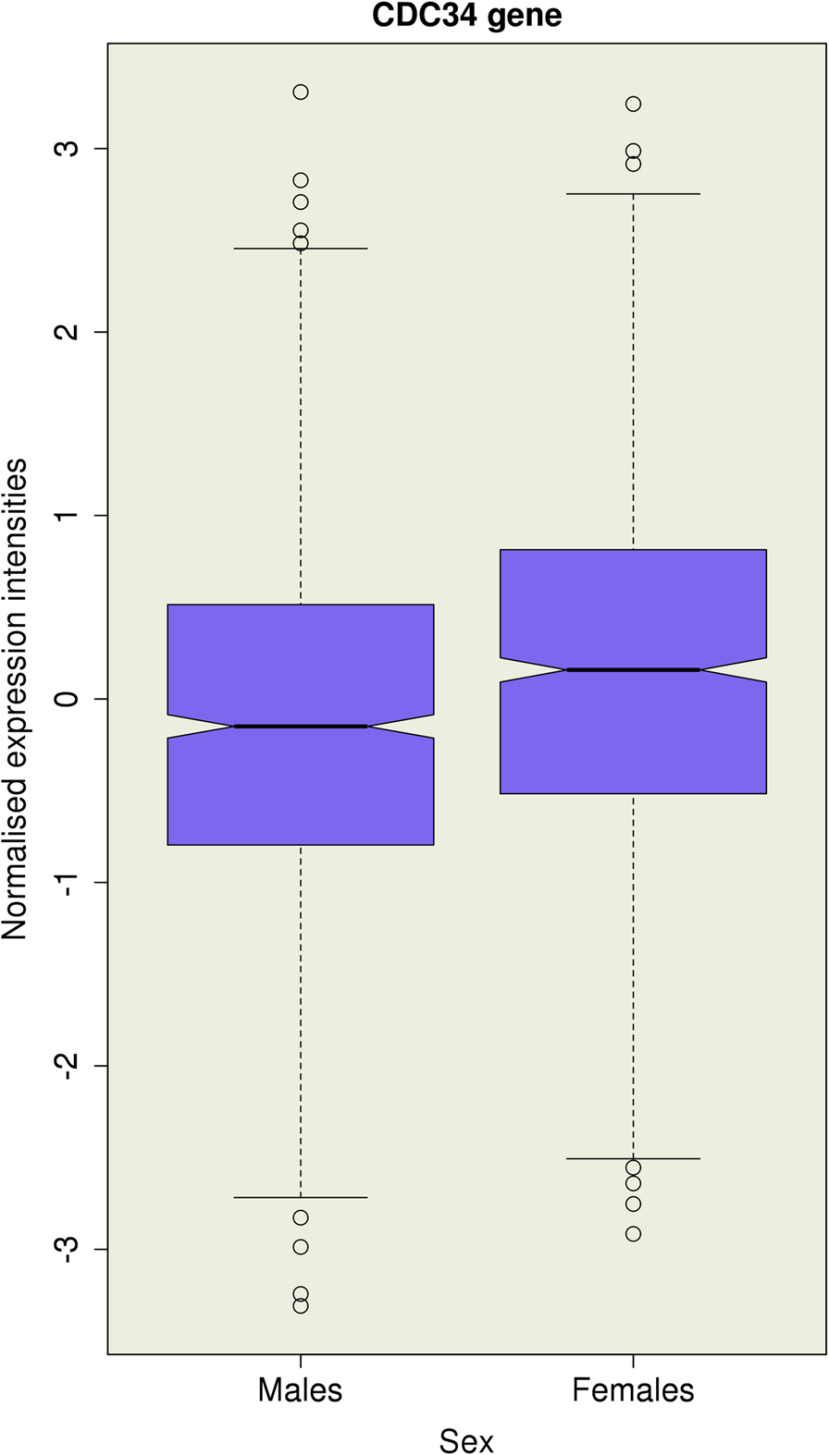
Distribution of normalised gene expression intensities of *CDC34* for *n*=1048 males and *n*=1005 females. Higher gene expression intensities are observed in females compared to males (*P*=3.66×10^−11^)

The bivariate GREML method [23] implemented in the GCTA software [24] was then used to estimate the genetic correlation of these 12,528 gene expression probes across the sexes captured by 796,005 imputed autosomal HapMap3 SNPs. The bivariate GREML method allows us to treat each gene expression probe as a distinct trait for males and females from which genetic correlations are estimated (see ‘Methods’). Each estimate was tested for deviation from *r*_G_=1, which indicates that the autosomal genetic control of gene expression differs across the sexes. A total of 28 of these analyses did not converge and were discarded. The quantile–quantile plot for expected versus observed *P* values from a likelihood ratio test is illustrated in Fig. 1. As shown, the distribution of *P* values is initially flat with values of 0.5, which is attributed to the likelihood ratio test being on the edge of the parameter space (see ‘Methods’) [25]. Subsequently, the distribution closely follows the null distribution with little deviation of the test statistics from the expected (genomic control, *λ*_GC_=1.05). The left panel of Fig. 2 illustrates the distribution of the estimated *r*_G_. As shown, the distribution is skewed towards 1, with a large peak at values close to 1. The median estimate across all tested probes is *r*_G_=1.00 indicating that, on average, males and females share the same common genetic control of gene expression. The right panel of Fig. 2 compares the estimated *r*_G_ with their corresponding *P* value. As shown, no probes satisfied the Bonferroni corrected significance threshold of *P*=3.99×10^−6^, which accounts for the number of probes tested. The smallest *P* value corresponded to the cell division cycle 34 (*CDC34*) gene on chromosome 19 with estimated *r*_G_=0.36 (*P*=8.45×10^−6^) (Table 1). *CDC34* encodes a protein that is a part of a large multiprotein complex that is required for ubiquitin-mediated degradation of cell cycle G1 regulators, and for the initiation of DNA replication. As cell counts may differ across the sexes, we also adjusted the bivariate GREML model for a mixture of predicted and measured cell-count proportions (see ‘Methods’). In this analysis, the *CDC34* gene deviated further from *r*_G_=1 with estimated *r*_G_=0.33 (*P*=2.24×10^−6^), which satisfied the Bonferroni significance threshold. A test for sexual dimorphism in gene expression at the *CDC34* gene revealed higher gene expression intensities in females compared to males ($\hat {\beta }=0.284$, SE =0.043; *P*=3.66×10^−11^) (Fig. 3).

**Table 1:** Ten most nominally significant probes from a bivariate GREML analysis testing genetic correlations that deviate from *r*_G_=1

Probe	Chr.	Position	Gene	$h^{2}_{\text {mal}}$	$h^{2}_{\text {fem}}$	*r*_G_	SE	*P*
ILMN1713006	19	492686-492735	CDC34	0.68	0.64	0.36	0.14	8.45×10^−6^
ILMN2383058	20	1610083–1610132	SIRPG	0.68	0.54	0.27	0.15	3.54×10^−5^
ILMN1710017	17	59361234–59361283	CD79B	0.62	0.64	0.32	0.14	3.59×10^−5^
ILMN1796165	14	95080536–95080585	GLRX5	0.76	0.66	0.47	0.13	4.44×10^−5^
ILMN1715169	6	32654825–32654845	HLA-DRB1	0.94	0.86	0.72	0.08	1.23×10^−4^
ILMN1723520	1	156494456–156494505	CD1A	0.38	0.60	0.001	0.19	1.26×10^−4^
ILMN1675483	2	241418872–241418921	ANKMY1	0.67	0.70	0.53	0.13	1.64×10^−4^
ILMN1742001	1	145696009–145696058	CD160	0.70	0.67	0.42	0.13	1.75×10^−4^
ILMN1776998	15	76361232–76361281	DNAJA4	0.51	0.66	0.36	0.16	2.05×10^−4^
ILMN1662451	19	7659893–7659942	FCER2	0.74	0.70	0.47	0.13	2.15×10^−4^

No probes satisfied the Bonferroni corrected significance threshold of *P*=3.99×10^−6^. The parameters $h^{2}_{\text {mal}}$ and $h^{2}_{\text {fem}}$ represent the estimated heritability for males and females, respectively

*Chr.* Chromosome

**Fig. 4:**
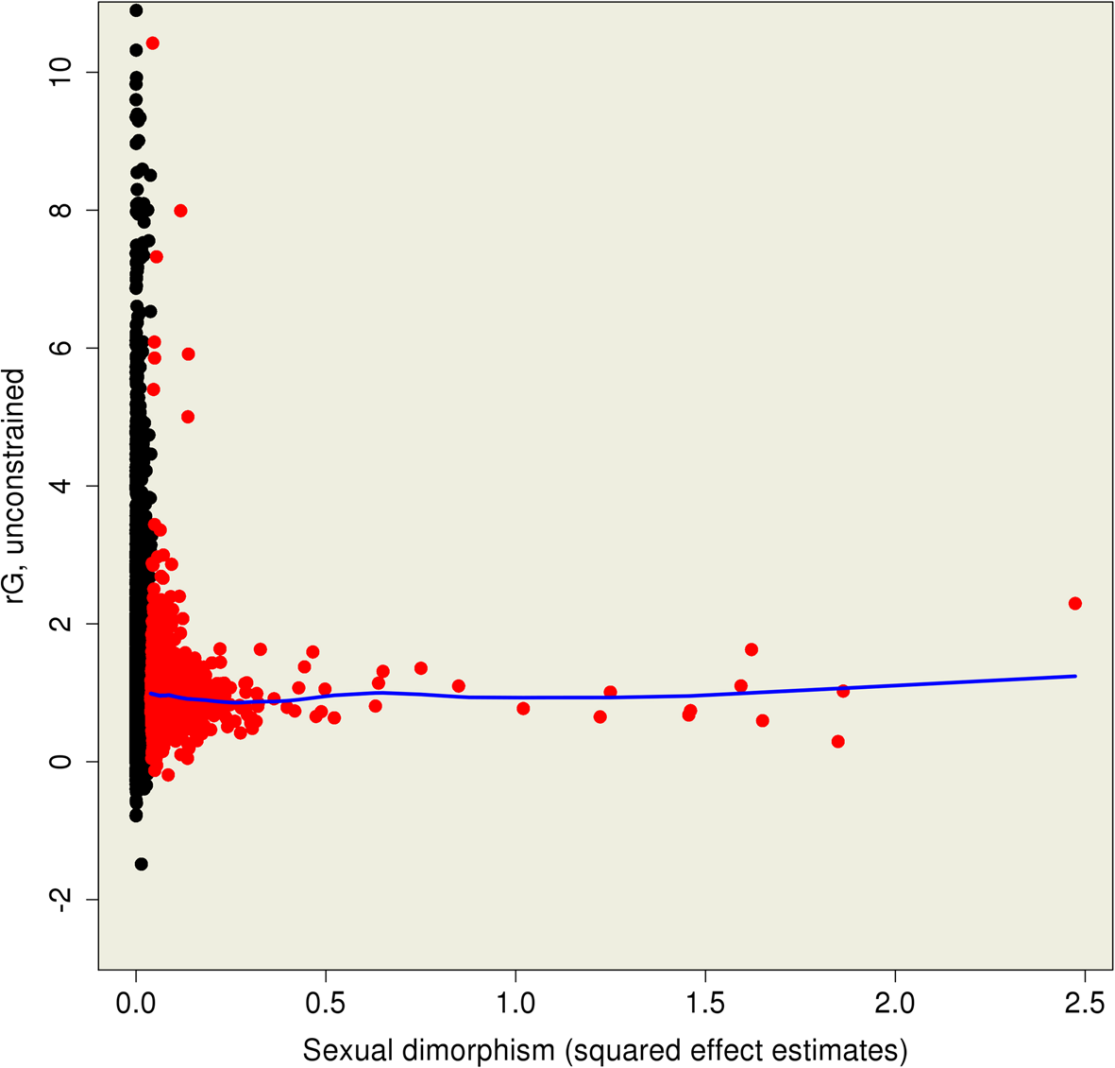
No trend was observed when comparing unconstrained estimates of *r*_G_ versus squared mean differences in gene expression across the sexes. The *red dots* are 1413 autosomal probes that showed significant mean differences in gene expression across the sexes. The *blue line* is a lowess line. Unconstrained *r*_G_ estimates were constrained to −2.5 to 10.5 to ease visualisation

We performed additional sensitivity analyses, including an unconstrained bivariate GREML analysis that gives unbiased estimates of *r*_G_ by allowing the estimates to go beyond the parameter boundary [−1,1]. Additional file 1: Figure S2 illustrates the distribution of the unconstrained estimates of *r*_G_, which had median *r*_G_=1.01 across all tested probes. We did not observe any trends when examining the relationship between unconstrained estimates of *r*_G_ and mean differences in gene expression across the sexes (Fig. 4). This is consistent with results from the study of the sex-specific genetic architecture of high-level human complex traits [26], but is in contrast to the observation of a negative relationship in a review of other multiple species, which found that traits with large mean phenotypic differences across the sexes had small or negative genetic correlations [16].

To test if there was common functionality in the 100 probes with the most nominally significant deviation from *r*_G_=1, we performed a gene ontology (GO) analysis using the DAVID functional annotation tool. This tested for significantly enriched biological process (BP) terms, molecular function (MF) terms and cellular component (CC) terms using a significance threshold of *P*<0.01 [27, 28]. There were no MF or CC terms with *P*<0.01. There was weak enrichment for BP terms, with the top terms corresponding to *immune response* (GO:0006955; *P*=2.00×10^−3^) and *regulation of multi-organism process* (GO:0043900; *P*=0.01); however, these terms did not survive a correction for multiple testing.

## Discussion and conclusions

This study examined the sex-specific genetic architecture of gene expression by estimating the genetic correlation of gene expression across the sexes. We first confirmed extensive sexual dimorphism in gene expression by demonstrating that 1413 autosomal probes corresponding to 1266 unique genes showed significant mean differences in gene expression intensities across the sexes. Such results have been the motivation for sex-stratified GWAS or genotype-by-sex interaction studies. Despite this, however, the median of *r*_G_ estimates across all tested probes is approximately 1, indicating that males and females share the same common genetic control of gene expression in whole blood. We did not observe any trends when examining the relationship between unconstrained estimates of *r*_G_ and mean differences in gene expression across the sexes, which is consistent with results from the study of sex-specific autosomal genetic architecture of high-level human complex traits [26], but is in contrast to the negative relationship observed in a study of other species [16]. Finally, a GO analysis revealed that the 100 probes with the most nominally significant deviation from *r*_G_=1 were weakly enriched for *immune response* and *regulation of multi-organism process* biological process terms with *P*<0.01, but did not survive a correction for multiple testing. The results from this study are broadly consistent with those observed in an examination of sex-specific autosomal genetic architecture of high-level human complex traits, which found estimated genetic correlations to be large and positive, indicating that males and females share the same common genetic control of these traits [13, 14].

These results address the weak evidence found in the literature for sex-specific eQTLs. In particular, they point to the possibility that the contribution of genotype-by-sex interactions to variation in gene expression may occur in a relatively small number of genes, and on average, males and females share the same common genetic control of gene expression. This is consistent with the limited findings in the literature for sex-specific eQTLs where, for example, a study with a relatively large sample size of *n*=922 individuals detected six sex-specific eQTLs [19], and a study with a relatively small sample size of *n*=390 individuals was not able to detect sex-specific eQTLs [18]. This suggests that eQTLs that harbour true sex-specific effects may occur in a small number of genes and without sufficiently large sample sizes, the power to detect them can be low. An outlier in the literature is a study that claims that 15% of detected *cis*-eQTLs show sex-specific effects, which corresponds to approximately 200 *cis*-eQTLs [17]. Individuals included in this study were from four HapMap populations (*n*≈100 individuals in each), with each population stratified by sex for the analysis (approximately *n*≈50 males and *n*≈50 females in each population). Therefore, the power to detect sex-specific *cis*-eQTLs in this scenario would likely be low given the sample sizes, and indeed, false discovery rates in this study were reported to be approximately 20%. Thus, the fraction of eQTLs that harbour true sex-specific effects in this study is likely to be small.

Drawing parallels between the study of the sex-specific genetic architecture of gene expression and high-level complex traits may give us further insight into the interpretation of our results. Like the study of sex-specific eQTLs, demonstrating sex-specific genetic effects in high-level complex traits has been challenging and largely unsuccessful due to the lack of power to robustly detect genotype-by-sex interactions [26]. Two recent studies examined the sex-specific genetic architecture of height, BMI and other high-level complex traits by estimating genetic correlations across the sexes [13, 14]. If we consider the results for height and BMI, it was shown that by doubling the sample size from *n*≈50,000 individuals to *n*≈100,000 individuals, there is a gain in the accuracy of the *r*_G_ estimates for these traits, but that the estimates remain large and positive, indicating that males and females share the same common genetic control for these traits. Importantly, it was also shown that <1% of the phenotypic variance for height and BMI can be explained by incorporating sex-specific genetic effects [14]. Taken together, these results indicate that the contribution of genotype-by-sex interaction to variation in these traits is relatively small compared to the main effect, and will thus require very large sample sizes to detect them. That is, results from these analyses do not rule out individual sex-specific genetic effects, but, broadly, they diminish the importance of genotype-by-sex interactions in the study of high-level complex traits [26]. The true power of this study does not come from individual tests for deviations from *r*_G_=1, but from estimating genetic correlations across approximately 13,000 traits, allowing us to examine the distribution of *r*_G_ estimates. Like the study of high-level complex traits, this analysis does not rule out individual sex-specific genetic effects, but, given the weak evidence for sex-specific eQTLs and the distribution of *r*_G_ estimates skewed towards 1, these results are consistent with the conclusion that the contribution of genotype-by-sex interactions to variation in gene expression is small and may occur in a relatively small number of genes. Further, we can postulate that with an increase in sample size, we can expect a corresponding increase in accuracy for the estimates of genetic correlation in gene expression across the sexes, but on average, these estimates will remain large and positive.

A limitation of the interpretation of our results is that gene expression intensities were measured in whole blood. Since sexually dimorphic genes have been shown to have tissue-specific patterns [20–22], it is possible that we may observe differences in the genetic control of gene expression across sexes if expression intensities are measured in other tissues. For example, if we measure gene expression in brain tissue, we may observe more cases where the genetic control of the expression of genes related to neurological and psychiatric disorders differs across the sexes. Conversely, despite being an appropriate tissue, results from our analysis in whole blood did not detect differences in the genetic control of the expression of genes related to autoimmune diseases. Indeed, it would be of interest to examine the distribution of *r*_G_ estimates across other tissue types, and in particular, if there is a significant shift away from *r*_G_=1; however, large sample sizes in a variety of different tissue types would be required for comprehensive investigation. One further limitation is that the genetic correlations were estimated based on common imputed HapMap3 SNPs Minor allele frequency (MAF) >0.01, but it is possible that rare variants of large effect may individually have a different effect in males and females, making them better discriminators between the sexes. However, because common SNPs imputed to 1000 Genomes capture the majority of genetic variation [29], it is unlikely that the aggregate effect of rare variants would significantly shift the distribution of *r*_G_ estimates away from 1. That is, it is unlikely that the cumulative effect of this missing part of the genetic (co)variance matrix would decrease the estimates of genetic correlation. Indeed, this was observed by Rawlik et al., where estimates of genetic correlation using common SNPs and a combined set of common and rare SNPs had a correlation of 0.98 across 19 complex traits [14]. Future work could explore this in more detail; however, a comprehensive analysis would require a considerably larger sample size and additional methodological work to overcome the inherent bias in the estimates with the inclusion of rare variants [29].

In conclusion, this study shows that the combined additive genetic effects of all SNPs across the autosomal chromosomes have the same effect on gene expression measured in whole blood in both sexes. These results are consistent with previous studies of sexual dimorphism in high-level complex traits in humans.

## Methods

### Study participants

Gene expression and genotype data were available in 2058 individuals from three distinct cohorts. Briefly, the Brisbane Systems Genetics Study (BSGS) is a family-based study comprising 846 individuals of Northern European origin from 312 independent families [30, 31]. Families consisted of adolescent monozygotic (MZ) or dizygotic (DZ) twins, their siblings and parents. RNA was collected from whole-blood samples with expression levels measured in 47,323 genome-wide probes using the Illumina HumanHT-12 v4.0 Beadchip. Individuals were genotyped using the Illumina 610-Quad Beadchip. Following standard quality control (QC) filtering, 528,509 SNPs were available for analysis.

The Coronary Artery Disease (CAD) cohort comprised 147 unrelated individuals enrolled in the Emory Cardiovascular Biobank, USA, with suspected or confirmed CAD [32]. RNA was collected from whole-blood samples with expression levels measured in 47,231 genome-wide probes using the Illumina HumanHT-12 Beadchip. Individuals were genotyped using the Illumina OmniQuad arrays, with 707,046 SNPs available for analysis.

Finally, the Estonian Genome Centre, University of Tartu (EGCUT) cohort consisted of 1065 unrelated individuals from Estonia [33]. RNA was collected from whole-blood samples with expression levels measured in 48,803 genome-wide probes using the Illumina HumanHT-12 v3.0 Beadchip. Altogether, 903 unique individuals were genotyped using the Illumina HumanCNV array and 162 unique individuals were genotyped using Human OmniExpress-12 v1.0. A total of 335,036 and 710,831 SNPs were available for analysis from each genotype dataset, respectively.

These gene expression and genotype datasets were concatenated following the QC procedures described below.

### Gene expression normalisation and quality control

Gene expression normalisation was first carried out on the individual gene expression datasets before concatenation. Variance stabilisation was applied using the method of Huber et al. [34] using the Bioconductor vsn package, followed by quantile normalisation. To account for known procedural variances (i.e. batch effects) in the BSGS cohort, we regressed gene expression levels for each probe on the chip ID, position on the chip and extraction date. Residuals from this analysis were carried forward as the corrected expression levels. Similarly, for the CAD and EGCUT cohorts, we regressed gene expression levels for each probe on the first ten principal components (PCs) and used the residuals as the corrected expression levels for each cohort [35]. We verified that sex effects were not removed from these corrected expression levels in the CAD and EGCUT cohorts by examining the correlation between the ten PCs and sex for each cohort; that is, for each PC, we used a *t*-test to test for mean differences across the sexes in each cohort, with a significant difference indicating that the corresponding PC removed sex effects from gene expression intensities. We did not detect significant differences in the ten PCs across the sexes, indicating that sex effects were not removed during PC correction (results not shown). A rank normal transformation was applied to each gene expression dataset to further standardise the gene expression levels. We concatenated these gene expression datasets by retaining a total of 38,624 probes that were common to all cohorts.

To avoid false positive results due to technical artefacts generated by cross-reactivity, we tested 36,951 autosomal gene expression probes for cross-hybridisation with X and/or Y chromosomes using BLAST [36]. Probes were classified as cross-hybridising with sex chromosomes if their sequences had 90% identity over the aligned region, at least 40 of 50 matching bps, and no gaps. A total of 598 cross-hybridising probes were excluded. Additionally, we filtered 24,702 probes with estimated heritability less than 10%, 134 probes that were not significantly expressed above background variation, 198 probes that were not well characterised, 429 probes on the X chromosome and 35 probes on the Y chromosome. A total of 12,528 gene expression probes targeting 10,274 genes on the autosome were available for analysis.

### Genotype imputation and quality control

We imputed autosomal genotype data for each cohort by first estimating haplotypes using HAPI-UR: HAPlotype Inference for UnRelated samples, Version 1.01 [37]. Haplotype estimates were then passed to IMPUTE2 [38] for imputation to 1000 Genomes Phase 1, Version 3. Following imputation, each genotype dataset contained approximately 38 million autosomal SNPs. The total number of SNPs was reduced to 5,398,402 by removing SNPs with an info score threshold of less than 0.9 [29], and by retaining SNPs that were common to all datasets. PLINK [39] was used to merge the datasets to form the final concatenated genotype dataset. Approximately 500 SNPs were excluded due to multi-allelic differences between cohorts. SNPs were excluded from the concatenated genotype dataset with Minor allele frequency (MAF) <0.01 and Hardy–Weinberg equilibrium test *P*<10^−6^ leaving 5,373,355 autosomal SNPs. We retained 796,005 autosomal HapMap3 SNPs that were common in the concatenated dataset to calculate a genetic relatedness matrix (GRM).

Five individuals from the CAD cohort showed evidence of non-European ancestry from multidimensional scaling analysis and were excluded. A total of 2053 individuals were available for analyses.

### Predicting cell counts

The proportion of neutrophils, lymphocytes and monocytes were predicted for individuals in the BSGS (*n*=223), CAD (*n*=142) and EGCUT (*n*=1065) cohorts from a deconvolution method proposed in [40] using raw gene expression intensities from 38,624 probes. Predicted cell-count proportions were obtained using the *gedBlood* command and the standard least squares regression approach in the CellMix package in R [41]. The method was first validated in the *n*=623 individuals in the BSGS cohort where measured cell-count proportions were available (Additional file 1: Figure S3).

### Sexually dimorphic gene expression

Differences in gene expression across the sexes were examined using a mixed linear regression model implemented in GCTA [24] to model gene expression levels as a linear function of male and female status. This can be written as

1 $$ \boldsymbol{y}_{\boldsymbol{i}} = \boldsymbol{a}_{\boldsymbol{i}} + {\boldsymbol{Xb}}_{\boldsymbol{i}} + \boldsymbol{g} + \boldsymbol{e}_{\boldsymbol{i}}  $$

where ***y*** is an *n*×1 vector of gene expression levels for probe *i*; ***a*** is the mean expression; ***b*** is the effect estimate for a fixed sex covariate, ***X***, where males are coded 0 and females are coded 1; ***g***, a random component to capture the polygenic effect and sample structure in the data; and ***e*** is the residual. The coefficient ***b*** can be interpreted as the difference in gene expression levels between males and females. We used the Wald statistic, calculated as the square of the effect estimate divided by the square of the standard error, to assess significance. A *P* value was calculated from a *χ* ^2^-distribution with one degree of freedom.

### Bivariate GREML analysis

To remove the effect of the difference in proportions of males and females across the cohort, a rank normal transformation was first applied to the gene expression matrix for males and females separately within each cohort. The genetic correlation (*r*_G_) between males (m) and females (f) for each gene expression probe was defined as

2 $$ r_{\mathrm{G}}=\frac{\operatorname{cov}(\boldsymbol{g}_{\mathbf{m}}, \boldsymbol{g}_{\mathbf{f}})}{\sqrt{\operatorname{var}(\boldsymbol{g}_{\mathbf{m}})\operatorname{var}(\boldsymbol{g}_{\mathbf{f}})}}  $$

where cov(***g***_**m**_,***g***_**f**_) is the estimated genetic covariance of gene expression levels at the probe sites between males and females, and var(***g***_**m**_) and var(***g***_**f**_) are the estimated genetic variances of gene expression levels at the probe sites for males and females, respectively.

We used the bivariate GREML method [23] implemented in the GCTA software [24] to estimate the genetic variance of gene expression for males and females, and the genetic covariance of gene expression between males and females that can be captured by 796,005 autosomal HapMap3 SNPs. The linear mixed-effects models for each sex can be written as.,

3 $$ \boldsymbol{y}_{\mathbf{m}} = \boldsymbol{X}_{\mathbf{m}} \boldsymbol{b}_{\mathbf{m}} + \boldsymbol{g}_{\mathbf{m}} + \boldsymbol{e}_{\mathbf{m}}  $$

4 $$ \boldsymbol{y}_{\mathbf{f}} = \boldsymbol{X}_{\mathbf{f}} \boldsymbol{b}_{\mathbf{f}} + \boldsymbol{g}_{\mathbf{f}} + \boldsymbol{e}_{\mathbf{f}}  $$

where ***y***_**m**_ and ***y***_**f**_ are *n*×1 vectors of gene expression levels for males and females, respectively. For ***y***_**m**_, we designate all gene expression levels measured in females as missing; similarly for ***y***_**f**_, we designate all gene expression levels measured in males as missing. ***b*** are vectors of fixed effects, ***g*** are random polygenic effects, ***X*** is the incidence matrix for the effects of ***b*** and ***e*** are residuals for each of the models. The variance-covariance matrix was defined as

$$V= \left[ \begin{array}{cc} \boldsymbol{A}_{\mathbf{m}} \boldsymbol{\sigma}^{\mathbf{2}}_{\boldsymbol{g}_{\mathbf{m}}} + \boldsymbol{I} \sigma^{2}_{e_{\mathrm{m}}} & \boldsymbol{A}_{\boldsymbol{m,f}} \boldsymbol{\sigma}^{\mathbf{2}}_{\boldsymbol{g}_{\mathbf{m}} \boldsymbol{g}_{\mathbf{f}}} \\ \boldsymbol{A}_{\boldsymbol{m,f}} \boldsymbol{\sigma}^{\mathbf{2}}_{\boldsymbol{g}_{\mathbf{m}} \boldsymbol{g}_{\mathbf{f}}} & \boldsymbol{A}_{\mathbf{f}} \boldsymbol{\sigma}^{\mathbf{2}}_{\boldsymbol{g}_{\mathbf{f}}} + \boldsymbol{I} \sigma^{2}_{e_{\mathrm{f}}} \end{array}\right] $$

where ***A***_**m**_ and ***A***_**f**_ are GRMs for males and females, respectively, and ***A***_***m,f***_ is the GRM between males and females based on SNP information. ***I*** is the identity matrix. $\sigma ^{2}_{\mathrm {G}}$, ${\sigma ^{2}_{e}}$ and $\sigma ^{2}_{g_{\mathrm {m}}g_{\mathrm {f}}}$ are the genetic variance for each sex, residual variance for each sex and covariance between ***g***_**m**_ and ***g***_**f**_, respectively. *r*_G_ was calculated for each probe using Eq. 2 and was tested against the null hypothesis that the genetic correlation is fixed at *r*_G_=1. We used a likelihood ratio test statistic to assess significance and calculated the *P* value from a *χ* ^2^-distribution. Due to the test being on the edge of the parameter space, the likelihood ratio test statistic is distributed as a 50:50 mixture of a point mass at 0 and a ${\chi _{1}^{2}}$-distribution [25]. We used the Bonferroni method to account for multiple testing.

We also performed an additional unconstrained bivariate GREML analysis using the –reml-no-constrain command in GCTA to obtain an unbiased estimate of *r*_G_ by allowing the estimates to go beyond the parameter boundary [−1,1]. Here, the likelihood ratio test statistic was compared to a ${\chi _{1}^{2}}$-distribution to calculate a *P* value. The bivariate models were also adjusted for a mixture of predicted and measured cell-count proportions: three continuous fixed-effect covariates for neutrophils, lymphocytes and monocytes as described previously, using actual values for *n*=623 individuals in the BSGS cohort and predicted values for the remaining individuals.

### Functional and pathway enrichment analysis

We performed a GO analysis on the 100 most nominally significant genes showing deviation from *r*_G_=1 using the DAVID functional annotation tool. This tested for significantly enriched BP terms, MF terms and CC terms [27, 28]. We report the associated GO functional category and pathways with *P*<0.01. Multiple testing was accounted for with the Bonferroni method.

## Additional file

Additional file 1 Supplementary figures. (PDF 971 kb)
