# Supplementary material for: Autosomal genetic control of human gene expression does not differ across the sexes
Source: Genome Biol. 2016 Dec 1;17:248. doi: 10.1186/s13059-016-1111-0 (PMC5134098; doi:10.1186/s13059-016-1111-0)
Supplement: Additional file 1 — Supplementary figures. (PDF 971 kb) [file 13059_2016_1111_MOESM1_ESM.pdf]

Autosomal genetic control of human gene expression does not differ across the sexes

Irfahan Kassam[1,*], Luke Lloyd-Jones[1], Alexander Holloway[1], Kerrin S. Small[2], Biao Zeng[3], Andrew Bakshi[1], Andres Metspalu[4], Greg Gibson[3], Tim D. Spector[2], Tonu Esko[4], Grant W. Montgomery[5,6], Joseph E. Powell[1,5], Jian Yang[1], Peter M. Visscher[1,8], and Allan F. McRae[1]

[1]Queensland Brain Institute, The University of Queensland, Brisbane 4072, QLD, Australia
[2]Department of Twin Research and Genetic Epidemiology, King's College London, London, United Kingdom
[3]School of Biology and Center for Integrative Genomics, Georgia Institute of Technology, Atlanta, GA 30332, USA
[4]Estonian Genome Center, University of Tartu, Tartu, Estonia
[5]Institute for Molecular Bioscience, University of Queensland, Saint Lucia, Brisbane, 4072, Australia
[6]Queensland Institute of Medical Research Berghofer Medical Research Institute, Brisbane 4029, QLD, Australia
[7]University of Queensland Diamantina Institute, Translational Research Institute, The University of Queensland, Brisbane 4072, QLD, Australia
[*]Correspondence: i.kassam@uq.edu.au

Keywords: Gene expression, genetic correlation, sex-specific genetic architecture

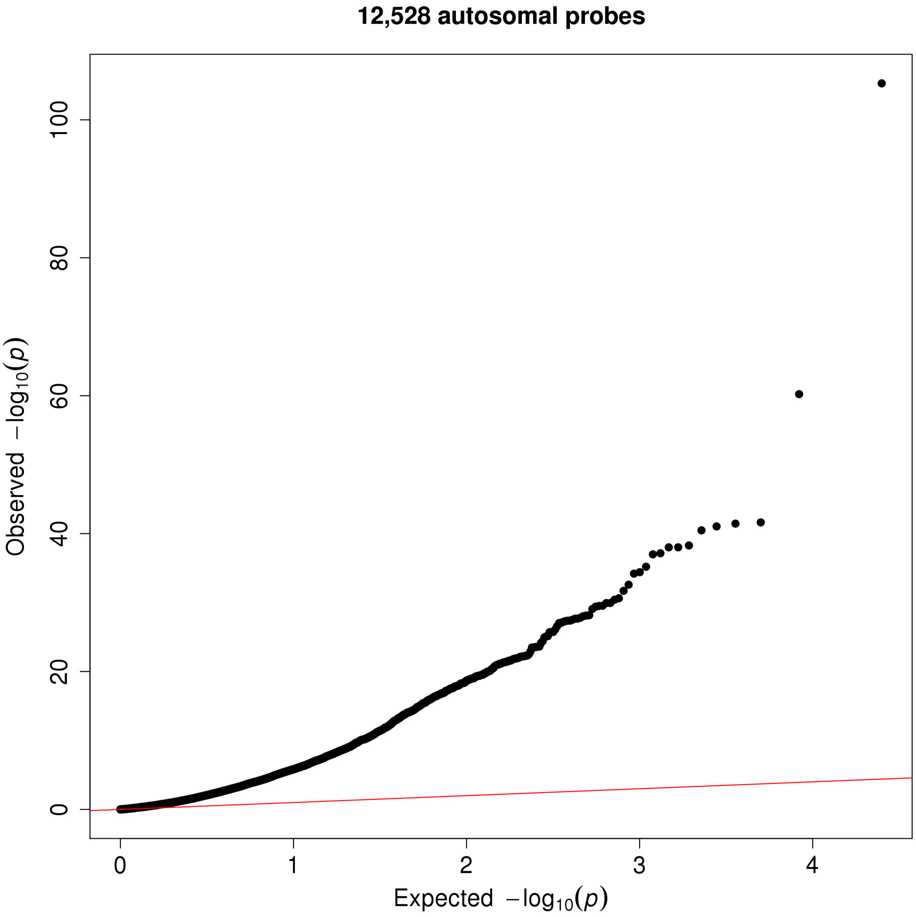

Figure S1: Quantile-quantile plot for expected versus observed P-values testing mean differences in gene expression intensities across the sexes for 12,528 autosomal probes. Extensive sexually dimorphic gene expression is illustrated by a large deviation of the observed test statistics from the expected. A total of 1,413 autosomal probes corresponding to 1,266 unique genes showed significant mean differences in expression intensities across the sexes at a Bonferroni corrected threshold of P $= 3.99 \times 10^{-6}$.

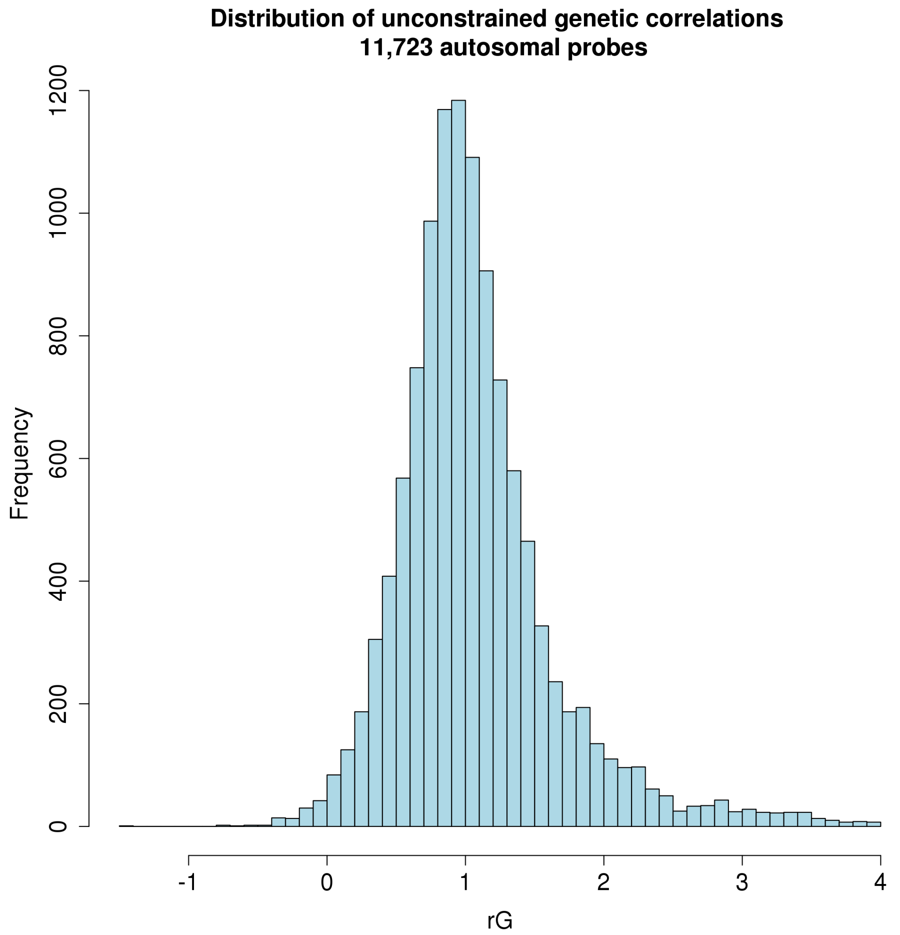

Figure S2: Distribution of 11,723 unconstrained estimates of genetic correlations of autosomal gene expression across males and females from a bivariate GREML analysis. The distribution had median $r_G = 1.01$ across all tested probes.

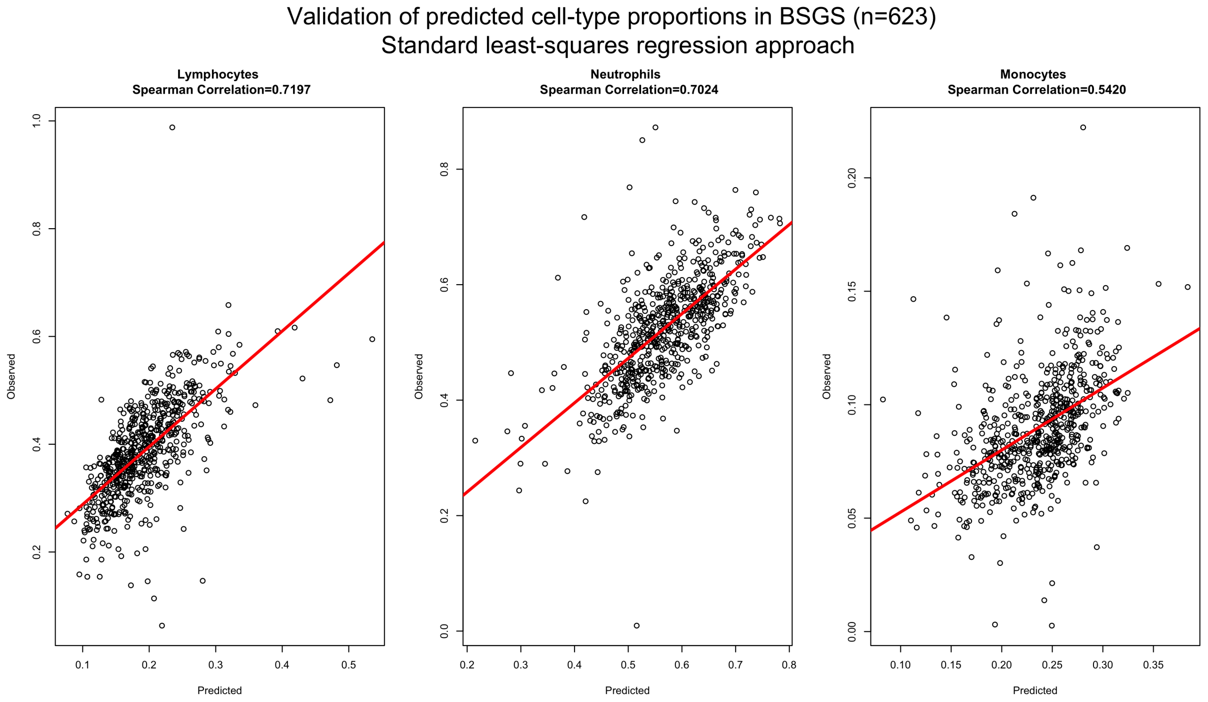

Figure S3: Predicted vs. observed cell-count proportions in $n = 623$ individuals from the BSGS cohort, validating the proposed deconvolution method [40].
